# Wnt/β-Catenin Signaling Regulates the Proliferation and Differentiation of Mesenchymal Progenitor Cells through the p53 Pathway

**DOI:** 10.1371/journal.pone.0097283

**Published:** 2014-05-12

**Authors:** Xu Peng, Liu Yang, Hongxing Chang, Gang Dai, Fuyou Wang, Xiaojun Duan, Lin Guo, Ying Zhang, Guangxing Chen

**Affiliations:** Center for Joint Surgery, Southwest Hospital, the Third Military Medical University, Chongqing, China; University of Alabama at Birmingham, United States of America

## Abstract

**Objective:**

Mesenchymal progenitor cells (MPCs) are found in articular cartilage from normal controls and patients with osteoarthritis (OA). Nevertheless, the molecular mechanisms of the proliferation and differentiation of these cells remain unclear. In this study, we aimed to determine the involvement of Wnt/β-catenin signaling in regulating the proliferation and differentiation of MPCs.

**Methods:**

MPCs were isolated from the articular cartilage of normal and OA patients. Cells were sorted by immunomagnetic cell separation. Cell proliferation capacity was evaluated using the MTT assay. Toluidine blue staining and immunostaining with anti-collagen II or anti-aggrecan antibodies were used to determine the chondrogenic differentiation capabilities of MPCs. The mRNA and protein expression of target genes were examined by quantitative real-time polymerase chain reaction and Western blotting, respectively. Knock-down of p53 expression was achieved with RNA interference.

**Results:**

Most cells isolated from the normal and OA patients were CD105^+^ and CD166^+^ positive (Normal subjects: CD105^+^/CD166^+^, 94.6%±1.1%; OA: CD105^+^/CD166^+^, 93.5%±1.1%). MPCs derived from OA subjects exhibited decreased differentiation capabilities and enhanced Wnt/β-catenin activity. Inhibition of Wnt/β-catenin signaling promoted proliferation and differentiation, whereas activation of this pathway by treatment with rWnt3a protein decreased the proliferation and differentiation of normal MPCs. Additionally, Wnt/β-catenin signaling positively regulated p53 expression, and silencing of p53 increased proliferation and differentiation of MPCs.

**Conclusions:**

Wnt/β-catenin regulated the proliferation and differentiation of MPCs through the p53 pathway.

## Introduction

Mesenchymal progenitor cells (MPCs), also known as mesenchymal stem cells, have been found in various human tissues, including human adult bone marrow [1]. These cells are thought to be involved in mesenchymal tissue maintenance and repair and may have great therapeutic potential due to their ability to self-renew and differentiate into multiple tissue types [2], [3]. When cultured *in vitro*, MPCs can be extensively expanded [4] and differentiated into multiple lineages, including bone, cartilage, tendon, muscle, nerve, and stromal cells, under specific conditions [5], [6]. A decline of MPC function has been suggested as a potential cause for the reduced ability of aged mammals to maintain mesenchymal tissue [2]. Nevertheless, the molecular mechanisms of MPC proliferation and differentiation are poorly understood.

Osteoarthritis (OA), a degenerative joint disease, is a major cause of pain and disability in adults [7]. MPCs have been identified in normal articular cartilage as well as OA cartilage. The frequency of MPCs is increased in OA cartilage [8], suggesting that these progenitor cells may be involved in the pathogenesis of arthritis. Notably, activation of β-catenin in articular chondrocytes in adult mice led to the development of an OA-like phenotype [9], and β-catenin levels are increased in human OA samples [9].

Brack *et al.* reported that Wnt signaling was increased in aging muscle stem cells, and injection of Wnt3A into young regenerating muscle led to decreased cellular proliferation and increased deposition of connective tissue [10]. Day *et al*. reported that the Wnt/β-catenin signaling pathway played a critical role in the differentiation of MPCs to osteoblasts and chondrocytes during vertebrate skeletogenesis, and conditional inactivation of β-catenin disrupted normal skeletal development [11]. The tumor suppressor p53 is closely associated with cell proliferation and differentiation, and it can induce apoptosis, cell cycle arrest, or senescence in response to stressors [12], [13]. The crosstalk between p53 and Wnt signaling pathways has been suggested by previous studies. For example, the downstream effector TCF-4 is been identified as a transcriptional target of p53 [14]. Overexpression of β-catenin in the cytoplasm of human retinoblastoma cells increased the transcriptional activity of p53 [15]. Therefore, p53 signaling plays a critical role in cellular aging, and Wnt signaling is closely associated with the p53 signaling pathway. We speculate that the Wnt signaling pathway may interact with p53 to regulate the aging of MPCs. In the present study, we explored the roles of the Wnt/β-catenin and p53 signaling pathways in the proliferation and differentiation of MPCs. Our findings may provide valuable insights into the pathogenesis of OA.

## Materials & Methods

### Reagents

Human Wnt3a recombinant protein (rWnt3a), secreted frizzled-related protein-3 (sFRP-3), and dickkopf-1 (Dkk-1) were purchased from R&D Systems (USA). The recombinant rhTGF-β3 protein was purchased from Peprotech (Rocky Hill, NJ, USA). Fetal bovine serum (FBS) and Eagle's minimal essential medium (MEM) were purchased from HyClone (South Logan, UT, USA). 3-(4,5-dimethylthiazol-2-yl)-2,5-diphenyltetrazolium bromide (MTT) was obtained from Sigma-Aldrich (St. Louis, MO). MicroBeads conjugated to monoclonal mouse anti-human CD105 and CD166, anti-mouse secondary antibodies coupled to magnetic beads, and primary antibodies anti-human CD105-PE, anti-human CD166-FITC, anti-mouse CD105, anti-mouse CD166 were obtained from Miltenyi Biotec (Germany), and anti-human β-catenin was obtained from Cell Signaling Technology (USA). Horseradish peroxidase (HRP)-conjugated goat anti-rabbit IgG was provided by Santa Cruz (USA). Trizol and Superscript III kits were purchased from Invitrogen (USA). The RNeasy mini kit was obtained from Qiagen (Netherlands).

### Sample collection

OA articular cartilage samples were collected from 10 patients (8 male) between 49.5 and 55.5 years old who were undergoing total knee arthroplasty from 2009 to 2011. In the OA group, articular cartilage from the femoral condyles was removed. In the control group, femoral head cartilage was collected from five subjects (4 male), age 58±5.2 years, undergoing total hip arthroplasty due to femoral neck fracture. The Ethics Committee of the Southwest Hospital approved the study protocols, and all participants provided written, informed consent prior to participation in the study. All samples were scored according to the Mankin scale, as described previously [16], [17].

### Cell culture

Cells isolated from articular cartilage were digested with type II collagenase (2 g/L) and trypsin (2.5 g/L). Cells were suspended in MEM supplemented with 10% FBS and seeded onto culture dishes at a density of 1×10^6^ cells/mL. Cells were maintained at 37 °C in a humidified environment containing 5% CO_2_ and 95% room air. The culture medium was change every 2 days.

### Immunomagnetic cell separation

When cell cultures reached 80% confluence, cells were digested, centrifuged, and resuspended in phosphate-buffered saline (PBS). Cells were incubated with 5 µL of magnetic microbeads conjugated to primary antibodies against CD105 and CD166 for 30 minutes, followed by secondary antibodies. The efficiency of magnetic separation was evaluated by flow cytometry. Positive and negative fractions were eluted with a double-sensitivity mode. Aliquots of CD105^+^ and CD166^+^ sorted cells were evaluated for purity by flow cytometry with a FACSCalibur machine (BD Biosciences).

### Immunocytochemical analysis

To characterize the isolated MPCs further, cells were labeled with anti-human CD105-PE and anti-human CD166-FITC conjugated antibodies and examined under a confocal laser microscope (Olympus IX70, Japan). To determine the chondrogenic differentiation capability of MPCs, cells were stained with toluidine blue (TB) or immunostained with anti-collagen II or anti-aggrecan antibodies.

### Wnt/β-catenin pathway inhibition and stimulation

Cells were randomly divided into four groups. Control cells were maintained in normal culture medium. The Wnt inhibition group was incubated with culture medium containing sFRP-3 (100 ng/mL) [18] and Dkk-1 (100 ng/mL) [19]. Cells in the Wnt stimulation group were treated with culture medium conditioned with rWnt3a (100 ng/mL) [20]. The Wnt neutralization group cells were incubated with medium containing sFRP-3 (100 ng/mL), Dkk-1 (100 ng/mL), and rWnt3a (100 ng/mL). To induce chondrogenic differentiation, cells were treated as described above for 4 days, and then incubated with culture medium supplemented with 1 mM sodium pyruvate, 10^–7^ dexamethasone, 1% ITS, 10 ng/mL rhTGF-β3, and antibiotics for 1 week.

### Quantitative real-time polymerase chain reaction (qRT-PCR)

Total RNA was extracted from cultured cells with Trizol reagent (MRC, USA). Total RNA was reverse-transcribed into cDNA using the First-strand cDNA Synthesis Kit (Fermentas), and qRT-PCR was carried out using the SYBR Green qPCR Mix (TOYOBO). Forward and reverse primers are listed in Table 1. The PCR reaction was initiated by incubation at 95 °C for 3 minutes, and samples were denatured at 95 °C for 15 seconds, annealed at 58 °C for 30 seconds, and extended at 72 °C for 40 cycles. For β-catenin, Sox9, and Wnt3a, the annealing temperature was 56 °C. The ratio of target gene expression to β-actin expression was calculated to normalize gene expression. Relative gene expression was calculated using the ΔΔC_t_ values, and results were expressed as 2^−ΔΔCt^ [21].

**Table 1:** PCR primers used to assess mRNA expression.

Target gene	Primers	Amplified length (bp)
Wnt3a	Forward: 5'-CCATCCTCTGCCTCAAATTC-3' Reverse: 5'-TGGACAGTGGATATAGCAGCA-3'	74
Wnt1	Forward: 5'-CACGACCTCGTCTACTTCGAC-3' Reverse: 5'-ACAGACACTCGTGCAGTACGC-3'	269
Wnt5a	Forward: 5'-ACACCTCTTTCCAAACAGGCC-3' Reverse: 5'-GGATTGTTAAACTCAACTCTC-3'	370
Collagen II	Forward: 5'-GCCTGGTGTCATGGGTTT-3' Reverse: 5'-GTCCCTTCTCACCAGCTTTG-3'	71
Aggrecan	Forward: 5'-AGGCTATGAGCAGTGTGAACG-3' Reverse: 5'-GCACGCCATAGGTCCTGA-3'	128
Sox9	Forward: 5'-TCCTCAGGCTTTGCGATTT-3' Reverse: 5'-TCCCAGCAGCACCGTTTT-3'	170
p53	Forward: 5’-CCCAAGCAATGGATGATTTGA-3’ Reverse: 5’-GGCATTCTGGGAGCTTCATCT-3’	91
β-actin	Forward: 5’-AGGGGCCGGACTCGTCATACT-3’ Reverse: 5’-GGCGGCACCACCATGTACCCT-3’	202

### Western blotting

Cells were washed with ice-cold PBS and incubated with lysis buffer (7 M urea, 2 M thiourea, 60 mM DTT, 4% CHAPS, 2% pharmalyte 3–10, and 1.4 mg/mL PMSF). The protein concentration was determined by Bradford assay. Total cell protein or cytosolic protein was separated by 10% sodium dodecyl sulfate-polyacrylamide gel electrophoresis and transferred to a polyvinylidene difluoride membrane. Membranes were blocked with 5% non-fat milk and then incubated successively with primary antibodies and HRP-conjugated secondary antibodies. The bound antibody complexes were detected using an enhanced chemiluminescence (ECL) reagent (Beyotime Institute of Biotechnology, Shanghai, China). The intensities of the immunoreactive bands were quantified by densitometry using IPP 6.0 software.

### Determination of cell proliferation

The MTT assay was used to examine cell proliferation. Cells were seeded on a 96-well plate at a density of 1×10^4^ cells/well. The next day, cells received different treatments. After a 24 hour incubation, cells were washed with PBS, and 200 µL of MTT (0.5 mg/mL diluted in culture medium) were added to each well. After 3 hours at 37 °C in the dark, the MTT solution was removed, and 200 µL of dimethyl sulfoxide (DMSO) were added to each well to solubilize the MTT metabolic product. The absorbance of the dissolved formazan was measured at 570 nm (A_570_) with a microplate reader (Multiscan MK3, Thermo).

### Lentivirus production, cell infection, and RNA interference (RNAi)

For RNAi, 4 oligonucleotide sequences targeting p53 (NM_000546) were used: siRNA1, 5'-CCATCCACTACAACTACAT-3'; siRNA2, 5'-CCACTGGATGGAGAATATT-3'; siRNA3, 5'-GACTCCAGTG GTAATCTAC-3'; siRNA4, 5'-GGCCTTGGAACTCAAGGAT-3'. In addition, a non-targeted control, 5'-TTCTCCGAACGTGTCACGT-3', was used. All sequences were flanked by *Age*I and *EcoR*I sites, which were used to ligate them into the lentiviral-based pMAGic 7.1 vector. Correct construction of recombinant products was confirmed by DNA sequencing. For virus production, human embryonic kidney 293T cells were seeded and transfected at 24 hours with the pRsv-REV, pMDlg-pRRE, and pMD2G plasmids. Viral supernatants were collected 48 hours after transfection and stored in aliquots at -80 °C. MPCs were incubated with viral particles for 24, 48, or 72 hours.

### Statistical analysis

Statistical analyses were performed using the SPSS 13.0 statistical software package. Data were presented as mean ± standard error [19]. Results were analyzed using one-way ANOVA with Fisher's Least Significant Difference (LSD) test. *P-*values <0.05 were considered statistically significant.

## Results

### Identification of isolated MPCs

**Figure 1:**
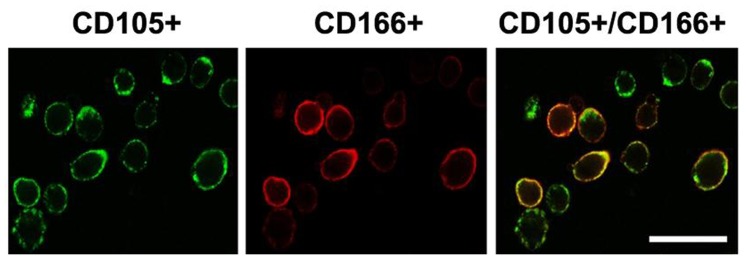
Immunocytochemical analyses of cells isolated from articular cartilage of OA subjects stained with CD105-PE and CD166-FITC. Samples were collected from 5 normal subjects and 10 OA patients. Data from 3 independent experiments were combined. Fluorescence was examined using a confocal laser microscope. Scale bar represents 50 µm.

Approximately 4.5% of the cells isolated from the normal and OA patient articular cartilage were CD105^+^ and CD166^+^ positive (Normal subjects: CD105^+^/CD166^+^, 4.6%±0.1%; OA patients: CD105^+^/CD166^+^, 4.5%±0.1%). Greater than 93% of the isolated cells from each group were viable (Normal subjects: CD105^+^/CD166^+^, 94.5%±1.5%; OA patients: CD105^+^/CD166^+^, 93.8%±1.2%). The proportion of cells expressing hematopoietic markers (CD34^+^ or CD45^+^) was less than 1% (Normal subjects: CD45^+^, 0.4%±0.0%; CD34^+^, 0.6%±0.0%; OA patients: CD45^+^, 0.5%±0.0%; CD34^+^, 0.8%±0.0%; *P*>0.05). Immunocytochemical analyses indicated that both CD105 and CD166 were expressed on the cell membranes (Figure 1), suggesting that cartilage from both groups contained MPCs.

### Characteristics of MPCs isolated from normal and OA articular cartilage

**Figure 2:**
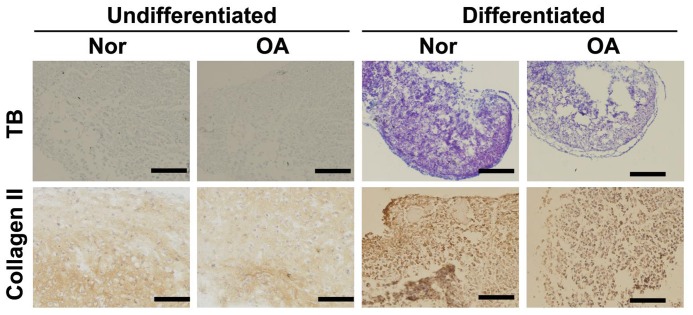
MPCs derived from normal (Nor) or OA subjects were cultured in normal culture medium (undifferentiated or induced to differentiate for 14 days (differentiated). Cells were stained with TB or labeled with anti-collagen II antibody. Samples were collected from 5 normal subjects and 10 OA patients. Data from 3 independent experiments were combined. Scale bar represents 200 µm.

Culture medium containing sodium pyruvate, dexamethasone, ITS, rhTGF-β3, and antibiotics was used to induce chondrogenic differentiation. Increased TB and collagen II staining results distinguished differentiated MPCs from undifferentiated cells (Figure 2). Differentiated MPCs derived from normal subjects showed more intense TB and collagen II staining than MPCs derived from OA subjects, suggesting that MPCs derived from OA patients were less differentiated (Figure 2).

### Wnt/β-catenin signaling in normal and OA human articular cartilage

**Figure 3:**
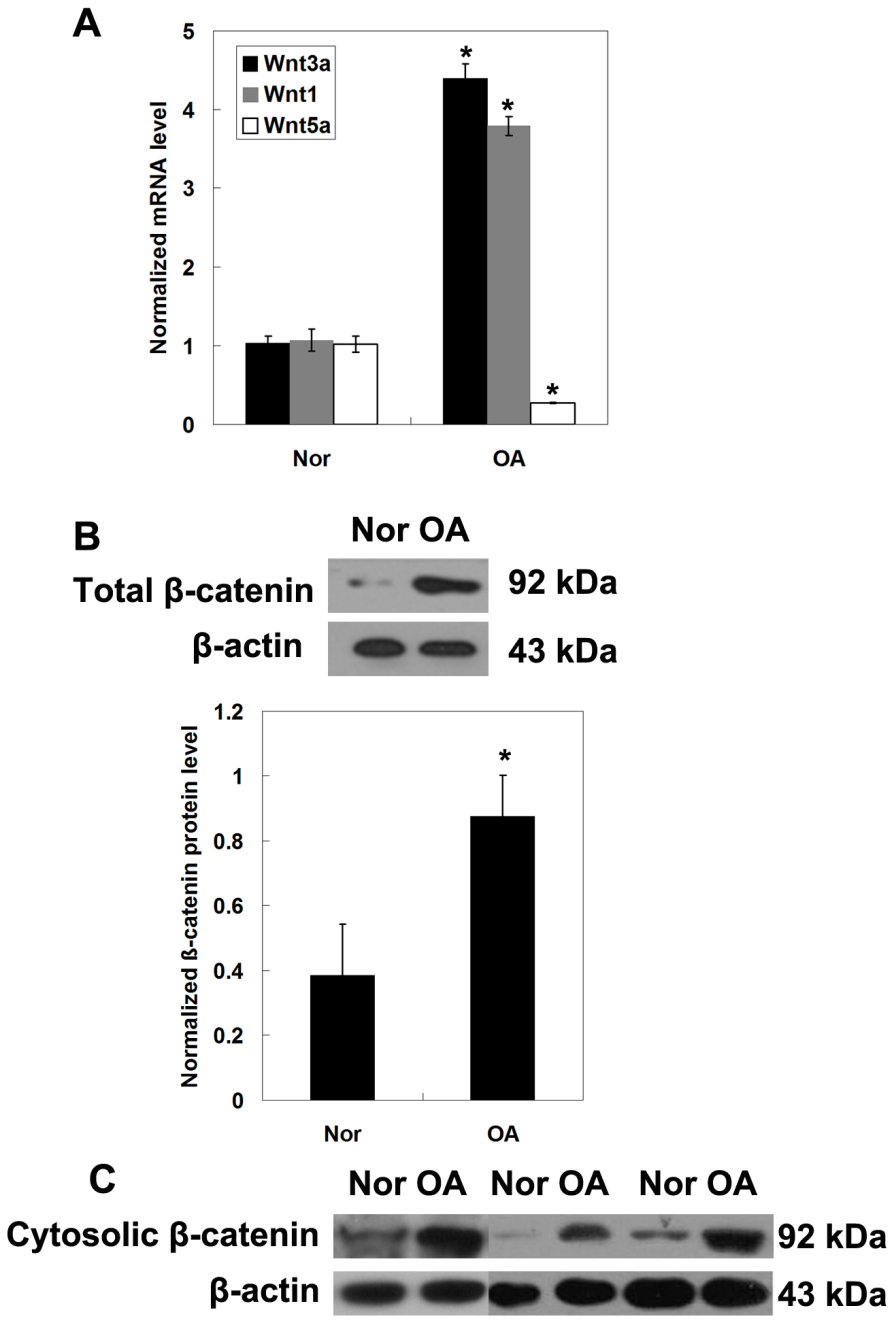
Expression of Wnt3a, Wnt1, and Wnt5a mRNA (A) and total (B) and cytosolic (C) β-catenin protein in MPCs of normal (Nor) and OA subjects determined by qRT-PCR and Western blotting, respectively. Data were collected from 3 independent experiments. Each experiment included samples collected from 5 normal subjects and 10 OA patients. Representative data are presented. *P<0.05 compared to normal control.

To investigate the involvement of the Wnt/β-catenin signaling pathway in MPCs derived from normal and OA human articular cartilage, the canonical Wnt pathway proteins Wnt1 and Wnt3a and the noncanonical Wnt pathway protein Wnt5, all of which play important roles during cartilage development and diseases, were examined [22]–[26]. Here, we observed increased mRNA expression of Wnt1 and Wnt3a (*P*<0.05), but decreased Wnt5 mRNA level in OA subjects as compared with control donors (*P*<0.05) (Figure 3A). In addition, the total and cytosolic protein expression of β-catenin was increased in MPCs of OA patients (*P*<0.05) (Figure 3B), suggesting that the Wnt/β-catenin signaling pathway activity was increased in MPCs of OA subjects.

### Effects of Wnt/β-catenin signaling on the proliferation and differentiation of MPCs

**Figure 4:**
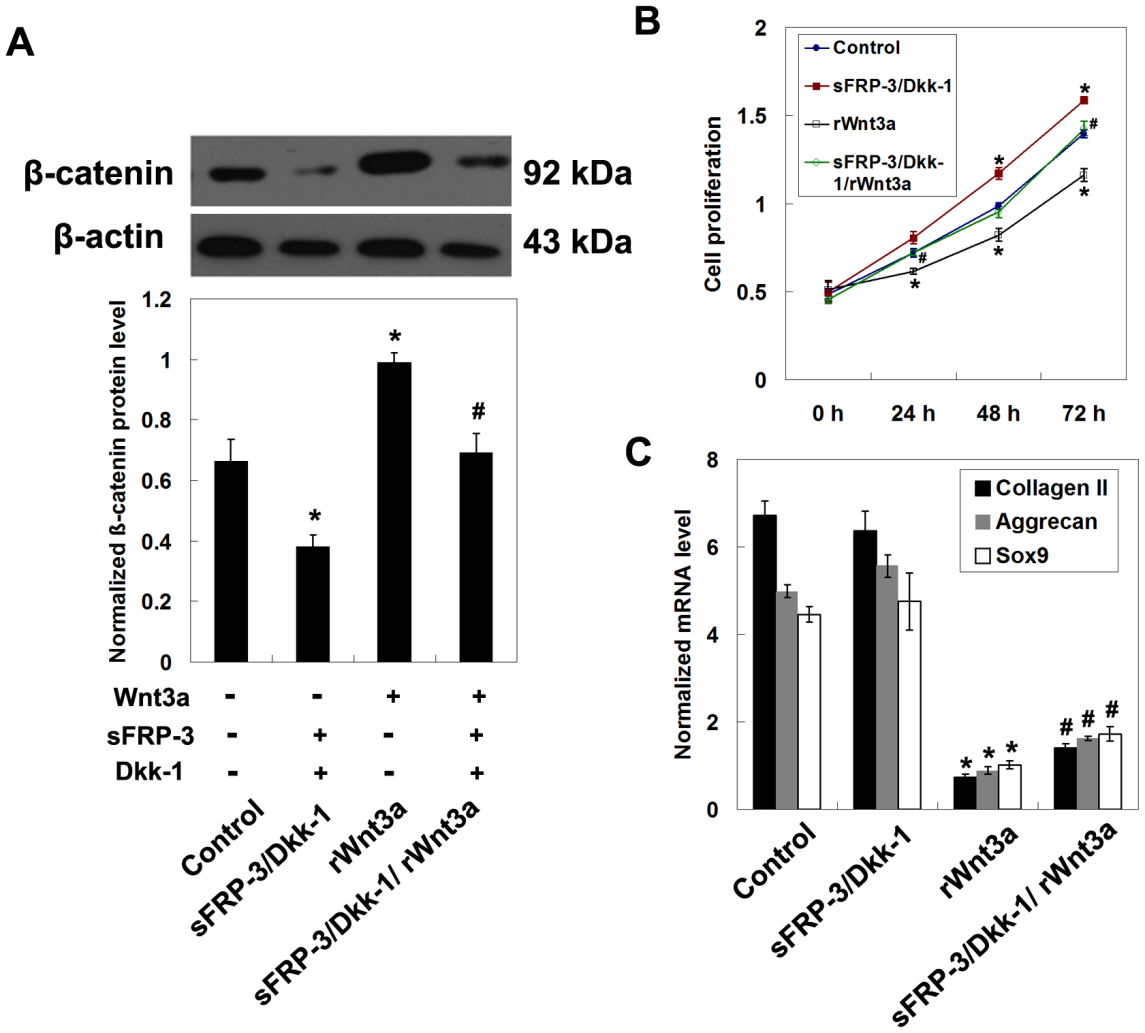
Influence of Wnt/β-catenin signaling on the proliferation and differentiation of MPCs. (A) Expression of β-catenin after 24 hours of treatment evaluated by Western blotting. (B) Cell proliferation at 24, 48, or 72 hours after treatment (*n* = 3). (C) Gene expression of collagen II, aggrecan, and SOX9 after differentiation evaluated by qRT-PCR (*n* = 3). Samples were collected from the 5 normal subjects. Data from 3 independent experiments were combined. *P<0.05 compared to control; #*P*<0.05 compared to rWnt3a.

We investigated the role of Wnt/β-catenin signaling in regulating the proliferation and differentiation of normal MPCs using Wnt3a inhibitors and rWnt3a. Inhibition of Wnt3a by treatment with sFRP-3 and Dkk-1 significantly reduced β-catenin protein levels, whereas treatment with rWnt3a increased β-catenin expression (Figure 4A). No difference was found when cells were treated with the Wnt3a inhibitors sFRP-3, Dkk-1, and rWnt3a together (*P*>0.05 compared with control). Cell proliferation was examined at 24, 48, and 72 hours after drug treatment. Cells treated with rWnt3a exhibited reduced cell proliferation compared to control (*P*<0.05 at 24, 48, and 72 hours) (Figure 4B). Significantly increased cell proliferation was observed in cells treated with sFRP-3 and Dkk-1 for 48 or 72 hours. No difference was found between control cells and cells incubated with sFRP-3, Dkk-1, and rWnt3a (*P*>0.05). In addition, treatment with rWnt3a reduced the mRNA levels of collagen II, aggrecan, and SOX9 (*P*<0.05 compared with control) (Figure 4C). These data suggest that the inhibition of Wnt/β-catenin promoted the proliferation and differentiation of MPCs.

### Effects of Wnt/β-catenin signaling on p53 expression in MPCs

**Figure 5:**
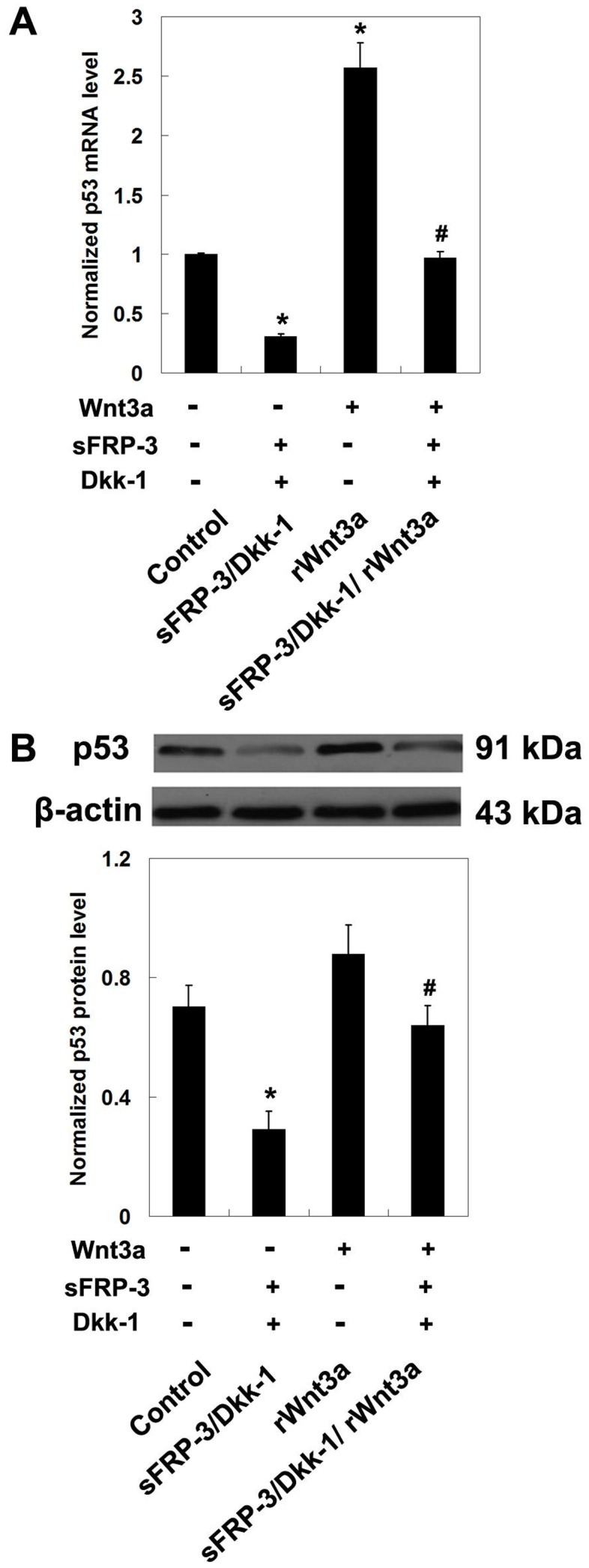
Influences of Wnt/β-catenin signaling on p53 expression. The mRNA (A) and protein expression (B) of p53 after 24 hours of treatment evaluated by qRT-PCR and Western blotting, respectively (*n* = 3). Samples were collected from 5 normal subjects. Data from 3 independent experiments were combined. *P<0.05 compared to control; #*P*<0.05 compared to rWnt3a treatment.

We also investigated the influences of Wnt/β-catenin signaling inhibition or stimulation on p53 expression. As shown in Figure 5, treatment with sFRP-3 and Dkk-1 decreased the mRNA and protein levels of p53 (*P*<0.05 compared with control), and stimulation of the Wnt signaling pathway with rWnt3a increased p53 mRNA and protein (*P*>0.05), implying that the Wnt/β-catenin signaling pathway positively regulated p53 expression in MPCs.

### Effects of p53 silencing on the proliferation and differentiation of MPCs

**Figure 6:**
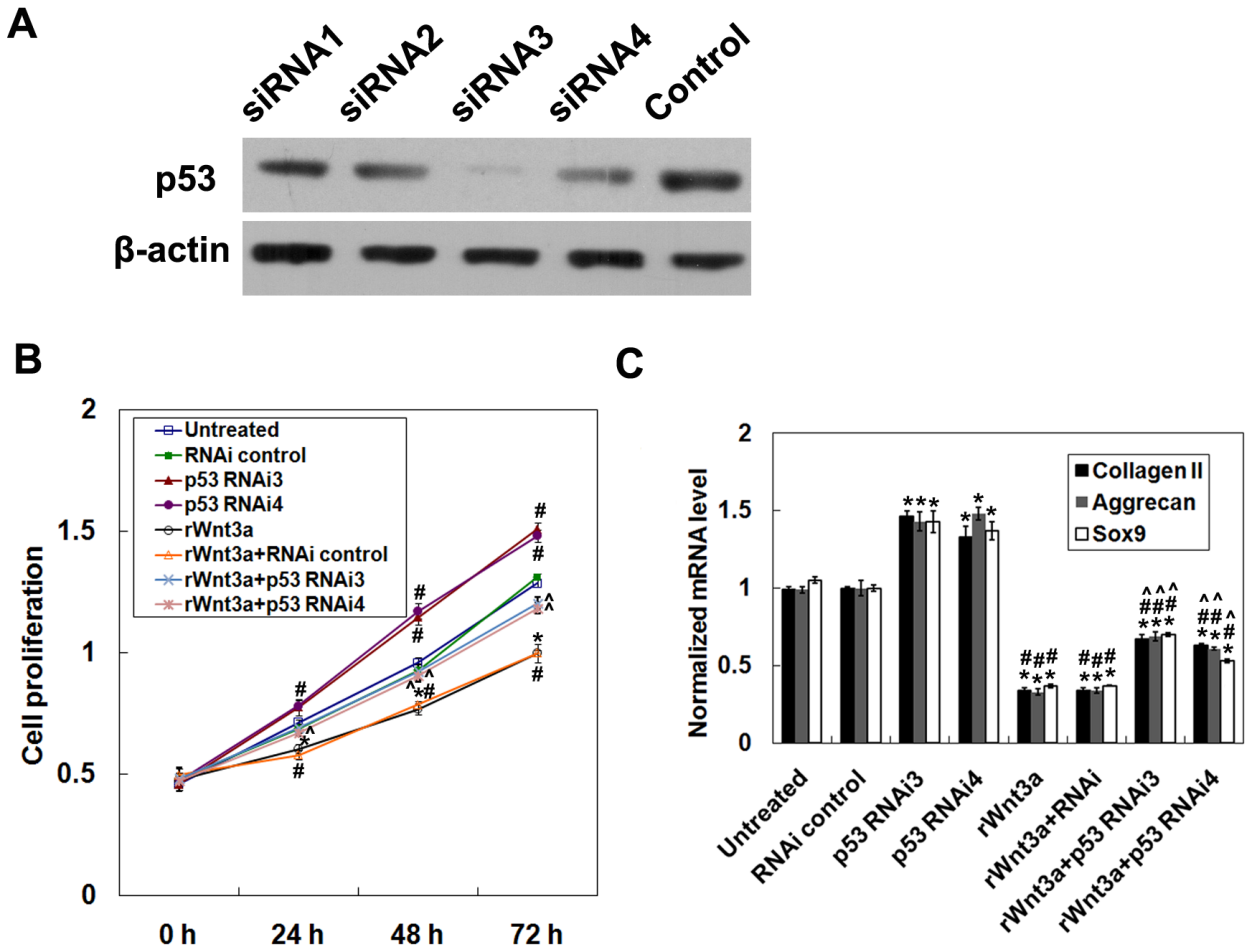
Effects of p53 silencing on the proliferation and differentiation of MPCs. (A) The siRNA knock-down efficiencies for p53. (B) Cell proliferation at 24, 48, and 72 hours after treatment (*n* = 3). * *P*<0.05 compared to normal control; # *P*<0.05 compared to p53 RNAi3; ∧ *P*<0.05 compared to p53 RNAi4. (C) mRNA levels of collagen II, aggrecan, and SOX9 evaluated by qRT-PCR (*n* = 3). Samples were collected from 5 normal subjects. Data from 3 independent experiments were combined. * *P*<0.05 rWnt3a vs. untreated; # *P*<0.05 compared to RNAi control; ∧ *P*<0.05 compared to rWnt3a+RNAi control.

To evaluate the effects of p53 on MPC proliferation and differentiation, we knocked down p53 using RNAi. Four siRNA sequences targeting p53 were designed. Western blot analyses using a specific p53 antibody showed that siRNA3 and siRNA4 efficiently down-regulated p53 expression (Figure 6A). Therefore, siRNA3 and siRNA4 were used in all subsequent experiments. As shown in Figure 6B, RNAi treatment increased cell proliferation as compared to the negative control (*P*<0.05 at 24, 48, and 72 hours), and incubation with rWnt3a decreased cell proliferation. In addition, treatment with RNAi and rWnt3a increased cell proliferation as compared with rWnt3a treatment alone (*P*<0.05). Knock-down of p53 with RNAi also increased the mRNA levels of collagen II, aggrecan, and SOX9 compared with control (*P*<0.05) (Figure 6C). In the presence of rWnt3a, the expression of these genes was reduced (*P*<0.05 compared with control), which could be partially reversed by silencing p53 with RNAi. These data suggest that downregulation of p53 can efficiently promote both cell proliferation and differentiation of MPCs.

## Discussion

We isolated MPCs from articular cartilage of normal and OA subjects. The percentage of MPCs in the articular cartilage was the same in both groups, in contrast with previous findings [8]. This discrepancy might be due to the limited sample size or the age of the OA patients included in the study (49.5–55.5 years). MPCs derived from OA subjects exhibited reduced ability to differentiate and enhanced Wnt/β-catenin activity.

Activation of β-catenin in articular chondrocytes in adult mice led to the development of OA-like phenotypes, including cell cloning, surface fibrillation, vertical clefting, and chondrophyte/osteophyte formation [9]. Notably, β-catenin levels were increased in human OA samples [9]. In a study of adult mice with conditional activation of β-catenin, an OA-like phenotype and increased expression of aggrecan in chondrocytes reported. Importantly, chondrocytes express aggrecan under normal conditions, and β-catenin stimulation leads to overexpression. Therefore, it has been suggested that β-catenin triggers the overexpression of aggrecan in articular chondrocytes and plays roles in the pathogenesis of OA. In the present study, MPCs, which maintain stem cell properties, were used. In their undifferentiated condition, the stimulation of β-catenin reduced aggrecan levels, suggesting the influences of β-catenin are different in differentiated and progenitor cells. Here, inhibition of Wnt/β-catenin signaling with sFRP-3 and Dkk-1 promoted proliferation and differentiation, and activation of the pathway with rWnt3a treatment decreased the proliferation and chondrogenic differentiation of normal MPCs. Interestingly, SFRP3 and DKK1 co-treatment did not fully suppress the effects of Wnt3a on gene repression, although it did block β-catenin protein stabilization. It is possible that SFRP3 and DKK1, in purified form, have reduced activity and are not expected to eliminate the gene regulatory effects completely. It is also possible that the stability of the target gene messages contributes to the existence of messages after β-catenin is destabilized. These data suggest that Wnt3a reduced the growth and differentiation of MPCs in OA subjects.

The tumor suppressor and transcriptional regulator p53 has been associated with apoptotic [27], autophagic [28], and necrotic cell death processes [29]. The p53 protein may act upstream of the canonical Wnt pathway to suppress its oncogenic activity [30]. In this study, we found that Wnt/β-catenin also positively regulated p53 expression, indicating that a feedback loop may regulate these two signals. In addition, silencing of p53 increased proliferation and differentiation of MPCs.

In summary, our current findings show that Wnt/β-catenin signaling regulated the proliferation and differentiation of MPCs through the p53 pathway. Future studies will investigate the detailed mechanism of the Wnt/β-catenin signaling pathway in the pathogenesis of OA using animal models.
